# New routes for spermine biosynthesis

**DOI:** 10.1016/j.jbc.2025.108390

**Published:** 2025-03-10

**Authors:** Bin Li, Hamid R. Baniasadi, Jue Liang, Margaret A. Phillips, Anthony J. Michael

**Affiliations:** Department of Biochemistry, UT Southwestern Medical Center, Dallas, Texas, USA

**Keywords:** bacteria, biosynthesis, polyamine, spermidine, spermine, thermospermine, *N*^1^-aminopropylagmatine, aspartate β-semialdehyde, carboxyspermidine, carboxyspermine, convergent evolution

## Abstract

The polyamine spermine (Spm) is a flexible linear teraamine found in bacteria and eukaryotes and in all known cases is synthesized from triamine spermidine by addition of an aminopropyl group acquired from decarboxylated *S*-adenosylmethionine (dcAdoMet). We have now identified in bacteria a second biosynthetic route for Spm based on the formation of carboxyspermine from spermidine, dependent on aspartate **β**-semialdehyde (ASA). This route also produces thermospermine (Tspm) from spermidine *via* carboxythermospermine. Two enzymes, carboxyspermidine dehydrogenase and carboxyspermidine decarboxylase, are responsible for ASA-dependent production of spermidine, Spm, and Tspm from diamine putrescine. Production of Spm/Tspm from spermidine is controlled primarily by carboxyspermidine dehydrogenase, not carboxyspermidine decarboxylase. This new ASA-dependent Spm biosynthetic pathway is an example of convergent evolution, employing nonanalogous, nonhomologous enzymes to produce the same biosynthetic products as the dcAdoMet-dependent Spm pathway. We have also identified bacteria that encode hybrid Spm biosynthetic pathways dependent on both dcAdoMet and ASA. In the hybrid pathways, spermidine is produced from agmatine primarily by the ASA-dependent route, and Spm is synthesized from agmatine or spermidine by dcAdoMet-dependent modules. Both parts of the hybrid pathway initiate from agmatine and each produces *N*^1^-aminopropylagmatine, so that agmatine, *N*^1^-aminopropylagmatine, and spermidine are common, potentially shared metabolites. Bacteria such as *Clostridium leptum* that encode the hybrid pathway may explain the origin of Spm produced by the gut microbiota. This is the first example of convergent evolution of hybrid dcAdoMet- and ASA-dependent *N*^1^-aminopropylagmatine, spermidine, and Spm biosynthesis encoded in the same genomes and suggests additional polyamine biosynthetic diversification remains to be discovered.

Spermine (Spm) phosphate was unwittingly first discovered by Leeuwenhoek in 1678, when he observed “glittering translucent” crystals in drying semen. These crystals were rediscovered in semen by Vacquelin in 1791 and by Böttcher in 1865 (reviewed by Rosenheim in 1924 (1)). In 1924, after successful isolation of ox pancreas-derived insoluble Spm phosphate crystals, the chemical composition of Spm was determined to be NH_2_(CH_2_)_3_NH(CH_2_)_4_NH(CH_2_)_3_NH_2_ (Fig. 1) (2). Biosynthesis of Spm was first confirmed in 1970 in rat prostate, where active Spm biosynthesis could not be separated from biosynthesis of the triamine spermidine, leading to the possibility that spermidine and Spm were synthesized by the same enzyme (Fig. 1) (3). However, soon after, using rat brain, it was found that spermidine biosynthesis could be separated from that of Spm, indicating distinct biosynthetic enzymes (4). Spermidine and Spm biosynthesis was shown to be dependent on the provision of aminopropyl groups from decarboxylated *S*-adenosylmethionine (dcAdoMet) (Fig. 1) (5). Production of spermidine was achieved by transfer of an aminopropyl group from dcAdoMet to putrescine by spermidine synthase, and Spm was formed by aminopropylation of the spermidine *N*^8^ amine, *i.e.*, the aminobutyl side of spermidine, by Spm synthase. The same mass structural isomer of Spm, thermospermine (Tspm), is formed by the dcAdoMet-dependent aminopropylation of the spermidine *N*^1^ amine, *i.e.*, the aminopropyl side of spermidine, by Tspm synthase (6) (Fig. 1).

**Figure 1:**
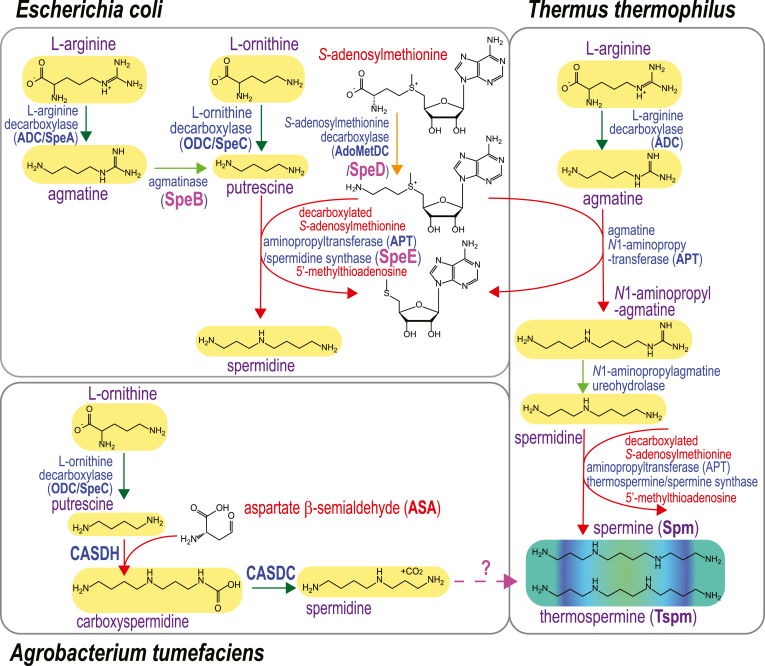
**Decarboxylated *S*-adenosylmethionine- and aspartate β-semialdehyde-dependent pathways for biosynthesis of spermidine and spermine/thermospermine in bacteria.** CASDH, carboxyspermidine dehydrogenase; CASDC, carboxyspermidine decarboxylase.

The specific functions of Spm in mammalian cells, *i.e.*, independent of spermidine, are difficult to determine unequivocally (7). This is because changes to Spm biosynthesis and catabolism affect the levels of spermidine and dcAdoMet due to feedback mechanisms. Spm is strongly associated with several aspects of development, including neural development, regulation of ion channels, and resistance to stress and reactive oxygen species (8). In the model flowering plant *Arabidopsis thaliana*, Spm is required for resistance to salt and drought stress (9, 10). In some flowering plants, Spm can be acylated by hydroxycinnamic acids, although the function of these Spm hydroxycinnamic amides is unknown (11). Spm is required for pantothenate and coenzyme A production in the yeast *Saccharomyces cerevisiae* (12). In bacteria, the function of Spm is unknown but it is often found in thermophilic species (13). Tspm is required for normal vascular development and growth in flowering plants (14), and Tspm and Tspm synthase are also found in phylogenetically diverse bacteria (13, 15).

The first cloning of a specific Spm synthase cDNA was from human cells, and the primary structure had little similarity to human spermidine synthase (16). The reason for the lack of similarity between the primary structures of human spermidine and Spm synthases was revealed by the X-ray crystal structure of the human Spm synthase, which showed that the N-terminal domain was derived from a bacterial class 1b *S*-adenosylmethionine decarboxylase (AdoMetDC) fused to a bacterial spermidine synthase-like domain (17, 18). In contrast, the plant Spm synthase is derived from a gene duplication of spermidine synthase followed by a change of substrate preference from putrescine to spermidine for one of the duplicated genes (19, 20). Similarly, the yeast Spm synthase is also derived from gene duplication of the yeast spermidine synthase (21). The dcAdoMet-dependent aminopropyltransferase (APT) family, besides spermidine and Spm synthases also includes Tspm synthase (6, 14) and agmatine *N*^1^-aminopropyltransferase (22, 23, 24) (Fig. 1).

Recently, phylogenetically diverse, dcAdoMet-dependent Spm, Tspm, spermidine, and *N*^1^-aminopropylagmatine synthases from bacteria were functionally identified (13). Spm was previously identified in the α-proteobacterial plant pathogen *Agrobacterium tumefaciens* (25). Induction of Spm accumulation in this species occurred upon growth in the presence of α-difluoromethylornithine, a suicide inhibitor of ornithine decarboxylase (ODC), which produces the spermidine biosynthetic precursor putrescine (Fig. 1) (26). Spm has also been found in the α-proteobacterium *Paracoccus denitrificans* after growth in low iron medium, which induces production of the spermidine-containing siderophore parabactin (27). These studies did not resolve Spm from the same mass isomer Tspm. However, unlike all known prokaryotic and eukaryotic species that produce Spm or Tspm, *A. tumefaciens* and *P. denitrificans* do not encode AdoMetDC or an APT. We sought to determine how Spm is synthesized in these species and functionally identified a new Spm/Tspm biosynthetic pathway dependent on aspartate β-semialdehyde (ASA) (Fig. 1), initiating from putrescine and utilizing carboxyspermidine and carboxyspermine (C-Spm)/carboxythermospermine (C-Tspm) intermediates. Furthermore, we identified phylogenetically diverse bacterial species that encode functional hybrid ASA-, dcAdoMet-dependent pathways, initiating from agmatine, that are specific for Spm biosynthesis.

## Results

### Spm in bacteria that do not encode APTs

The α-proteobacterium *A. tumefaciens* (Hyphomicrobiales order, now known as *Agrobacterium fabrum*) is a plant pathogen and is responsible for crown gall disease (28). This species does not encode AdoMetDC or an APT but does encode an alternative spermidine biosynthetic pathway consisting of carboxyspermidine dehydrogenase (CASDH) and carboxyspermidine decarboxylase (CASDC) (26, 29, 30) (Fig. 1). It also encodes a homospermidine synthase (WP_010973306; 481 a.a.). When grown in the presence of the ODC suicide inhibitor α-difluoromethylornithine, it accumulates Spm/Tspm. Twenty-five years previously, Spm/Tspm was detected in the α-protobacterium *P. denitrificans* (Rhodobacterales order), particularly during siderophore production (27). This species does not encode AdoMetDC or an APT but does encode CASDH and CASDC homologs. We hypothesized that Spm/Tspm may be synthesized through the CASDH/CASDC route and looked for other examples of bacteria that encode CASDH/CASDC homologs but not AdoMetDC and APT homologs and which have been found previously to accumulate Spm/Tspm.

Deep-sea hydrothermal vent bacteria, the ε-proteobacterium *Hydrogenimonas thermophila* and the Deferribacterota species *Deferribacter desulfuricans* were found to accumulate Spm/Tspm and encode CASDH and CASDC homologs (15, 31). Our genome analysis indicated that for *D. desulfuricans*, besides encoding CASDH and CASDC, it also encodes AdoMetDC and an APT. The facultatively psychrophilic γ-proteobacterium *Psychromonas marina* was found to accumulate Spm/Tspm, but Spm was not distinguished from Tspm (31). We also chose to investigate the CASDH/CASDC homologous enzymes from the α-proteobacterium *Brucella abortus* (Hyphomicrobiales order) because this species makes a spermidine-based, 2,3-dihydroxybenzoic acid containing siderophore brucebactin (32), similar in structure to agrobactin from *A. tumefaciens* and parabactin from *P. denitrificans* (33). This species also encodes a homospermidine synthase homolog (WP_346217511). We then investigated whether the CASDH/CASDC homologs from *A. tumefaciens*, *P. denitrificans*, *B. abortus*, *P. marina*, *H. thermophila, and D. desulfuricans* could produce spermidine and Spm/Tspm after coexpression in *Escherichia coli*.

### Spm is produced by an ASA-dependent pathway

**Figure 2:**
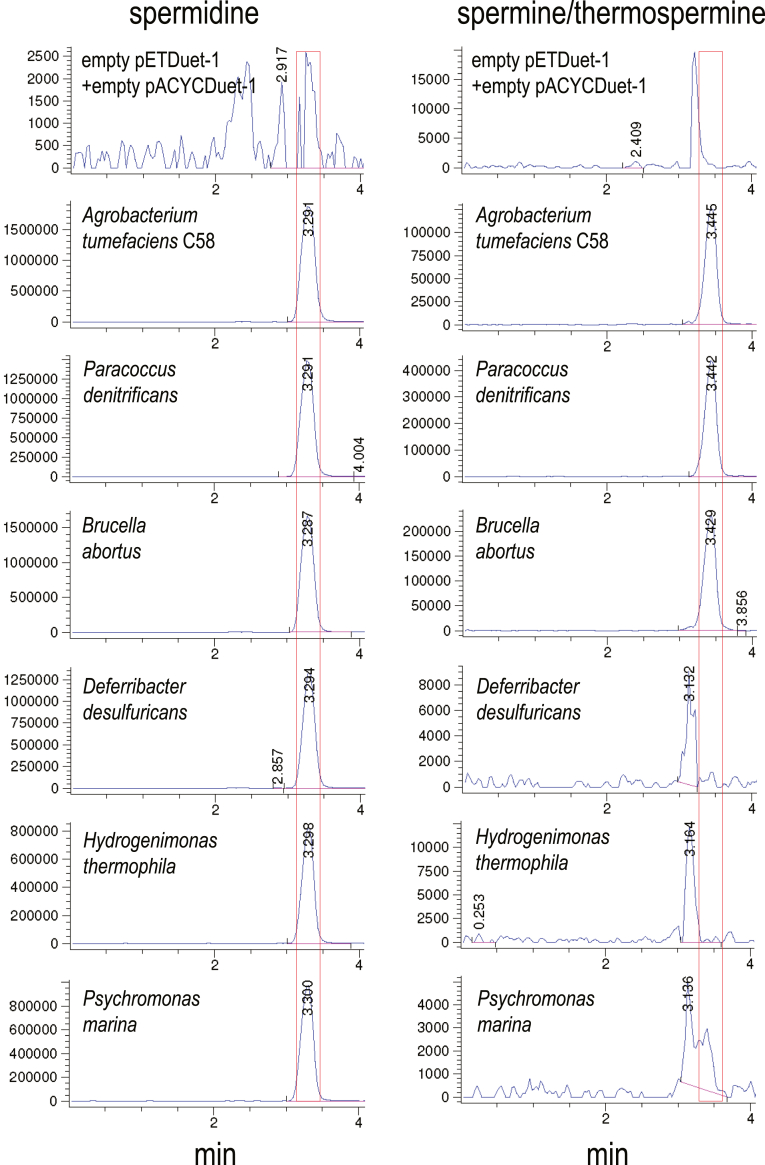
**Heterologous spermidine and spermine/thermospermine production by CASDH/CASDC homologs in *E. coli* BL21*speD*.** Polyamines from cell extracts were benzoylated and analyzed by LC-MS. The extracted ion chromatograms (EICs) for tribenzoylated spermidine (mass tolerance window 457.94:458.94) and tetrabenzoylated spermine/thermospermine (619.02:620.02) are shown. Carboxyspermidine dehydrogenase (CASDH) and carboxyspermidine decarboxylase (CASDC) homologs from the indicated species were coexpressed from pETDuet-1 and pACYCDuet-1, respectively, in spermidine-void *E.coli* BL21*speD* (AdoMetDC gene deletion). Spermidine or spermine/thermospermine peaks are highlighted by *red boxes*. The y-axis represents arbitrary units of ion intensity, and all samples were grown, extracted, and analyzed together. AdoMetDC, S-adenosylmethionine decarboxylase.

To assess the biosynthetic activity of the CASDH/CASDC pairs, we coexpressed the CASDH homologous gene in pETDuet-1 and the CASDC homologous gene in pACYCDuet-1 in a spermidine-void AdoMetDC gene deletion strain of *E. coli*, BL21*speD* (34). After growth of the *E. coli* strains in polyamine-free M9 medium and induction of gene expression, polyamines obtained from the cell extracts were benzoylated for analysis by LC-MS. This LC-MS approach does not effectively distinguish Spm from its same mass structural isomer Tspm. Fig. 2 shows that all CASDH/CASDC pairs were able to synthesize spermidine in the spermidine-void BL21*speD* cells. It is not possible to compare directly the relative biosynthetic efficiency of the different CASDH/CASDC pairs due to potential differences in steady-state protein levels, but there is at most a 2-fold difference in spermidine accumulated. In contrast to the pattern of spermidine accumulation, Spm/Tspm was produced only by the *A. tumefaciens*, *P. denitrificans,* and *B. abortus* CASDH/CASDC pairs, with a trace amount produced by the *P. marina* CASDH/CASDC. The level of Spm/Tspm produced by the *P. denitrificans* CASDH/CASDC pair was approximately 200-fold more than the corresponding *P. marina* enzymes. Ratios between spermidine to Spm/Tspm accumulation ranged from approximately 15:1 for the *A. tumefaciens* CASDH/CASDC to 3.1-fold for the *P. denitrificans* CASDH/CASDC. No detectable Spm/Tspm was produced by the *H. thermophila and D. desulfuricans* CASDH/CASDC homologs, but they did produce spermidine.

### Spm and Tspm are produced by the same enzymes

This LC-MS system does not discriminate well between Spm and Tspm; therefore, we analyzed the same but independently grown BL21*speD* strains coexpressing the *A. tumefaciens*, *P. denitrificans,* and *B. abortus* CASDH/CASDC genes, using LC-MS/MS analysis of the benzoylated polyamines, that we have previously demonstrated efficiently discriminates between Spm and Tspm (13). In addition, the CASDH/CASDC pairs were also expressed in the spermidine-replete spermidine *N*-acetyltransferase (SpeG) gene deletion mutant BL21*speG*, which is not capable of acetylating spermidine, Spm, or Tspm (34). Table 1 shows that both Spm and Tspm were produced by each CASDH/CASDC pair. The ratio between accumulated Spm to Tspm with each CASDH/CASDC pair in BL21*speD* was approximately 5:1 for *A. tumefaciens*, 8:1 for *P. denitrificans*, and 7:1 for *B. abortus*. The ratio between accumulated Spm to Tspm with each CASDH/CASDC pair in BL21*speG* was approximately 4:1 for *A. tumefaciens*, 3:1 for *P. denitrificans,* and 6:1 for *B. abortus*. This suggests that high spermidine in BL21*speG* increases the relative ratio of Tspm to Spm or that Spm is more efficiently *N*-acetylated by SpeG or both. Spm is formed from spermidine by aminopropylation of the *N*^8^-aminobutyl side of spermidine. The higher amount of Spm formed relative to Tspm suggests that the CASDH/CASDC pairs would synthesize *N*^1^-aminopropylhomospermidine from homospermidine, which possesses two aminobutyl groups, more efficiently than norspermine from norspermidine, which contains only aminopropyl groups. Consistent with this, approximately 5- to 8-fold more *N*^1^-aminopropylhomospermidine was produced by the *A. tumefaciens*, *P. denitrificans,* and *B. abortus* CASDH/CASDC pairs, compared to norspermine, when expressed in spermidine-void BL21*speD* grown with 500 μM homospermidine or norspermidine (Fig. S1). However, the relative uptake efficiencies of homospermidine and norspermidine are not known.

**Table 1:** LC-MS/MS analysis of spermine and thermospermine production by coexpression of carboxyspermidine dehydrogenase and decarboxylase in *E. coli* BL21*speD and* BL21*speG*

Experimental group/species	AUP 11.63 min (Spm)	AUP 10.92 min (Tspm)
*Group 1*, coexpression in *E. coli* BL21*speD*		
Empty pETDuet-1 + pACYCDuet-1	ND	ND
*Agrobacterium tumefaciens* CASDH + CASDC	2.98 × 10^6^	5.87 × 10^5^
*Paracoccus denitrificans* CASDH + CASDC	1.72 × 10^7^	2.25 × 10^6^
*Group 2*, co-expression in *E. coli* BL21*speG*		
Empty pETDuet-1 + pACYCDuet-1	1.07 × 10^5^	ND
*Agrobacterium tumefaciens* CASDH + CASDC	9.71 × 10^8^	3.59 × 10^8^
*Paracoccus denitrificans* CASDH + CASDC	1.04 × 10^9^	3.12 × 10^8^
*Group 3*, coexpression in *E. coli* BL21*speD*		
Empty pETDuet-1 + pACYCDuet-1	6.91 × 10^3^	ND
*Agrobacterium tumefaciens* CASDH + CASDC	1.99 × 10^8^	3.57 × 10^7^
*Brucella abortus* CASDH + CASDC	2.37 × 10^8^	3.61 × 10^7^
*Group 4*, coexpression in *E. coli* BL21*speG*		
Empty pETDuet-1 + pACYCDuet-1	2.74 × 10^4^	ND
*Agrobacterium tumefaciens* CASDH + CASDC	2.00 × 10^8^	3.84 × 10^7^
*Brucella abortus* CASDH + CASDC	3.08 × 10^8^	5.61 × 10^7^

CASDH expressed from pETDuet-1, CASDC from pACYCDuet-1. Strains in individual groups grown and analyzed together. Shown are AUP values for tetrabenzoylated spermine and thermospermine.

AUP, area under the peak (with elution time); ND, not detected; *speD*, gene deletion of *S*-adenosylmethionine decarboxylase; *speG*, gene deletion of spermidine *N*-acetyltransferase; Spm, spermine; Tspm, thermospermine.

### Carboxyspermidine, C-Spm, and C-Tspm intermediates

Biosynthesis of spermidine from putrescine by CASDH/CASDC proceeds *via* a carboxyspermidine intermediate (35, 36). We hypothesized that formation of Spm and Tspm from spermidine by CASDH/CASDC proceeds *via* a C-Spm and carboxy-Tspm (C-Tspm) intermediate. To test this hypothesis, we expressed the CASDH-encoding genes in either spermidine-void BL21*speD* or spermidine-replete BL21*speG* and used high-resolution LCMS to detect unbenzoylated carboxyspermidine (Table 2), C-Spm and C-Tspm (Table 3). Table 2 shows that in both BL21*speD* and BL21*speG*, the *A. tumefaciens*, *P. denitrificans* CASDH produced approximately 100-fold more carboxyspermidine than the *D. desulfuricans* and *H. thermophila* CASDH. The individual HR-LCMS chromatograms for carboxyspermidine are shown in Fig. S2. These data are surprising because all CASDH/CASDC pairs produced relatively similar amounts of spermidine in BL21*speD*, whereas the *A. tumefaciens* and *P. denitrificans* CASDH-encoding genes produced 100-fold more carboxyspermidine in BL21*speD* and BL21*speG* than the *D. desulfuricans* and *H. thermophila* CASDH homologs. This suggests that carboxyspermidine may not be an intermediate in spermidine biosynthesis by the *D. desulfuricans* and *H. thermophila* CASDH/CASDC homologous enzymes.

**Table 2:** High-resolution LCMS detection of carboxyspermidine produced by expression of CASDH homologs in *E. coli* BL21*speD* and BL21*speG*

Species	Identified mass at 2.5 min	Mass error (ppm)	AUP
*Expressed in BL21*speD			
Empty pETDuet-1	ND	ND	ND
*Agrobacterium tumefaciens* CASDH	190.1551	0.5	2.359 × 10^5^
*Paracoccus denitrificans* CASDH	190.1551	0.7	2.971 × 10^5^
*Deferribacter desulfuricans* CASDH	190.1545	−2.8	2.974 × 10^3^
*Hydrogenimonas thermophila* CASDH	190.1539	−5.7	3.284 × 10^3^
*Expressed in BL21*speG			
Empty pETDuet-1	ND	ND	ND
*Agrobacterium tumefaciens* CASDH	190.1552	1.1	2.915 × 10^5^
*Paracoccus denitrificans* CASDH	190.1550	0.2	2.634 × 10^5^
*Deferribacter desulfuricans* CASDH	190.1541	−4.9	4.874 × 10^3^
*Hydrogenimonas thermophila* CASDH	190.1548	−1.3	5.572 × 10^3^

HR-LCMS detection of underivatized *E. coli* cell extracts was performed in positive polarity mode after expression of pETDuet-1-based plasmids in either BL21*speD* or BL21*speG*. The theoretical average mass of carboxyspermidine is 189.255 Da. The identified carboxyspermidine precursor mass was 190.155 Da.

AUP, area under the peak (with elution time); CASDH, carboxyspermidine dehydrogenase; ND, not detected; *speD*, gene deletion of *S*-adenosylmethionine decarboxylase; *speG*, gene deletion of spermidine *N*-acetyltransferase.

**Table 3:** High-resolution LCMS detection of carboxyspermine/carboxythermospermine produced by expression of CASDH genes in *E. coli* BL21*speD* and BL21*speG*

Species	Identified mass at 6.85 min	Mass error (ppm)	AUP
*Expressed in BL21*speD			
Empty pETDuet-1	ND	ND	ND
*Agrobacterium tumefaciens* CASDH	ND	ND	ND
*Paracoccus denitrificans* CASDH	ND	ND	ND
*Deferribacter desulfuricans* CASDH	ND	ND	ND
*Hydrogenimonas thermophila* CASDH	ND	ND	ND
*Expressed in BL21*speG			
Empty pETDuet-1	ND	ND	ND
*Agrobacterium tumefaciens* CASDH	247.2126	−1.0	6.619 × 10^5^
*Paracoccus denitrificans* CASDH	247.2127	−0.5	9.817 × 10^5^
*Deferribacter desulfuricans* CASDH	ND	ND	ND
*Hydrogenimonas thermophila* CASDH	ND	ND	ND

HR-LCMS detection of underivatized *E. coli* cell extracts was performed in positive polarity mode after expression of pETDuet-1-based plasmids in either BL21*speD* or BL21*speG*. The theoretical average mass of carboxyspermine/carboxythermospermine is 246.35 Da. The identified carboxyspermine/carboxythermospermine precursor mass was 247.213 Da.

AUP, area under the peak (with elution time); CASDH, carboxyspermidine dehydrogenase; ND, not detected; *speD*, gene deletion of *S*-adenosylmethionine decarboxylase; *speG*, gene deletion of spermidine *N*-acetyltransferase.

**Figure 3:**
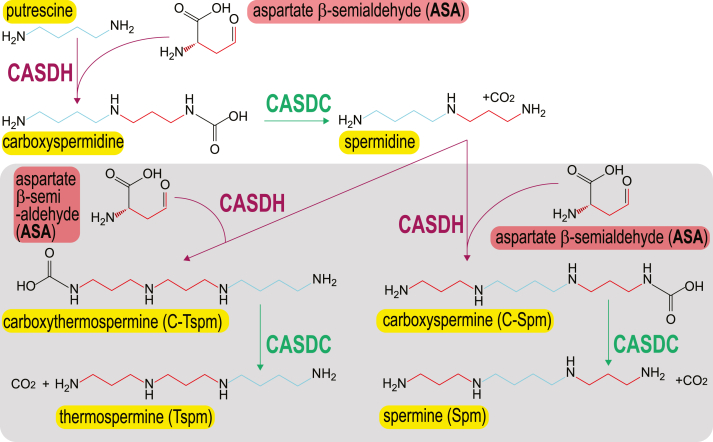
**A new pathway for spermine and thermospermine biosynthesis from putrescine *via* carboxyspermidine, carboxyspermine, and carboxythermospermine.** CASDH, carboxyspermidine dehydrogenase; CASDC, carboxyspermidine decarboxylase.

Table 3 shows detection of C-Spm/C-Tspm from the same samples, and in this system, C-Spm is not distinguished from C-Tspm. Individual C-Spm/C-Tspm HR-LCMS chromatograms are shown in Fig. S3. In spermidine-void BL21*speD*, none of the CASDH-encoding genes produced C-Spm/C-Tspm, presumably because carboxyspermidine is not decarboxylated to spermidine. However, in the spermidine-replete BL21*speG*, the CASDHs of *A. tumefaciens* and *P. denitrificans* efficiently produce C-Spm/C-Tspm. No detectable C-Spm/C-Tspm was produced by the *D. desulfuricans* and *H. thermophila* CASDHs. These data confirm that the production of Spm and Tspm by CASDH/CASDC proceeds *via* a C-Spm/C-Tspm intermediate (Fig. 3).

### A hybrid ASA- and dcAdoMet-dependent pathway for Spm biosynthesis

**Figure 4:**
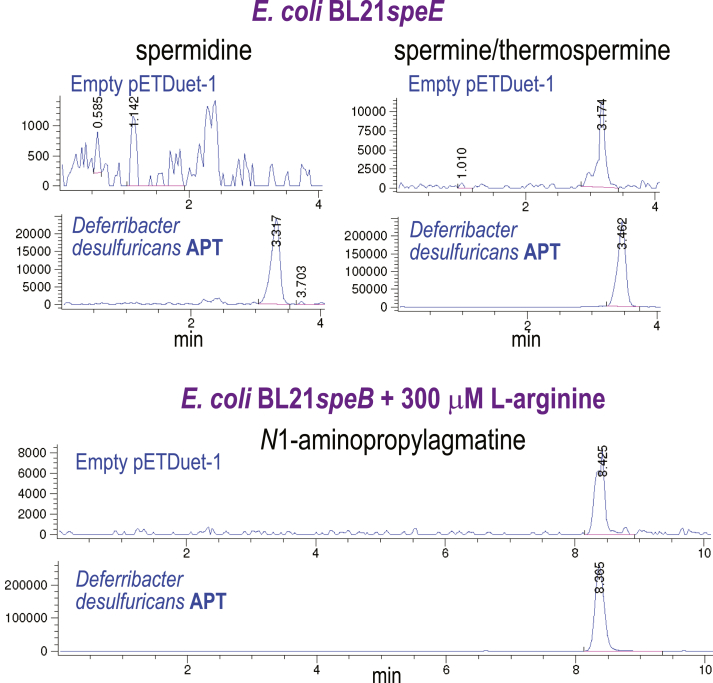
**Expression of the *Deferribacter desulfuricans* aminopropyltransferase in *E. coli* BL21*speE* and BL21*speB*.** The *D. desulfuricans* aminopropyltransferase (APT) was expressed from pETDuet-1 in either spermidine-void BL21*speE* or BL21*speB* grown with 300 μM L-arginine. Polyamines from cell extracts were benzoylated and analyzed by LC-MS. Shown are the extracted ion chromatograms for tribenzoylated spermidine (457.94:458.94), tetrabenzoylated spermine/thermospermine (619.02:620.02), and tetrabenzoylated *N*^1^-aminopropylagmatine (605:606). The y-axis represents arbitrary units of ion intensity, and all samples were grown and analyzed in parallel.

Previously, *D. desulfuricans* was found to accumulate Spm/Tspm at about 50% the level of spermidine (15), but coexpression of its encoded CASDH/CASDC pair produced spermidine but did not produce detectable Spm/Tspm (Fig. 2). We found that this species also encodes an AdoMetDC and an APT homolog; therefore, we investigated whether the APT could synthesize Spm/Tspm. Phylogenetically diverse APTs exhibit *N*^1^-aminopropylagmatine synthase activity (13). We therefore expressed the *D. desulfuricans* APT in a spermidine synthase gene deletion (Li 16) of *E. coli* (BL21*speE*) and in an agmatinase gene deletion strain BL21*speB* (13) to detect any spermidine, Spm/Tspm, or *N*^1^-aminopropylagmatine synthase activity by employing LC-MS for detection of benzoylated spermidine, *N*^1^-aminopropylagmatine, and Spm/Tspm (Fig. 4). When expressed in spermidine-void BL21*speE*, the APT produced a small amount of spermidine but at least 10-fold more Spm/Tspm. This is consistent with the behavior of other bacterial Spm/Tspm synthases, which are found in species that also encode separate spermidine/*N*^1^-aminopropylagmatine synthases (13). These data suggest that in *D. desulfuricans*, the CASDH/CASDH pair produce most of the spermidine, while AdoMetDC/APT produces the Spm/Tspm. This is the first demonstration that a functional hybrid ASA- and dcAdoMet-dependent polyamine metabolic pathway may exist in some bacteria. When expressed in BL21*speB* in the presence of 300 μM L-arginine to suppress ornithine production and increase agmatine accumulation, the *D. desulfuricans* APT produced *N*^1^-aminopropylagmatine at a level approximately 10-fold higher than it produced spermidine in BL21*speE*. These data strongly suggest that the *D. desulfuricans* APT initiates Spm/Tspm biosynthesis from agmatine *via N*^1^-aminopropylagmatine and spermidine. It is feasible that the AdoMetDC/APT biosynthetic module utilizes the spermidine produced by the CASDH/CASDC module.

### Hybrid Spm biosynthetic pathways in phylogenetically diverse bacteria

**Figure 5:**
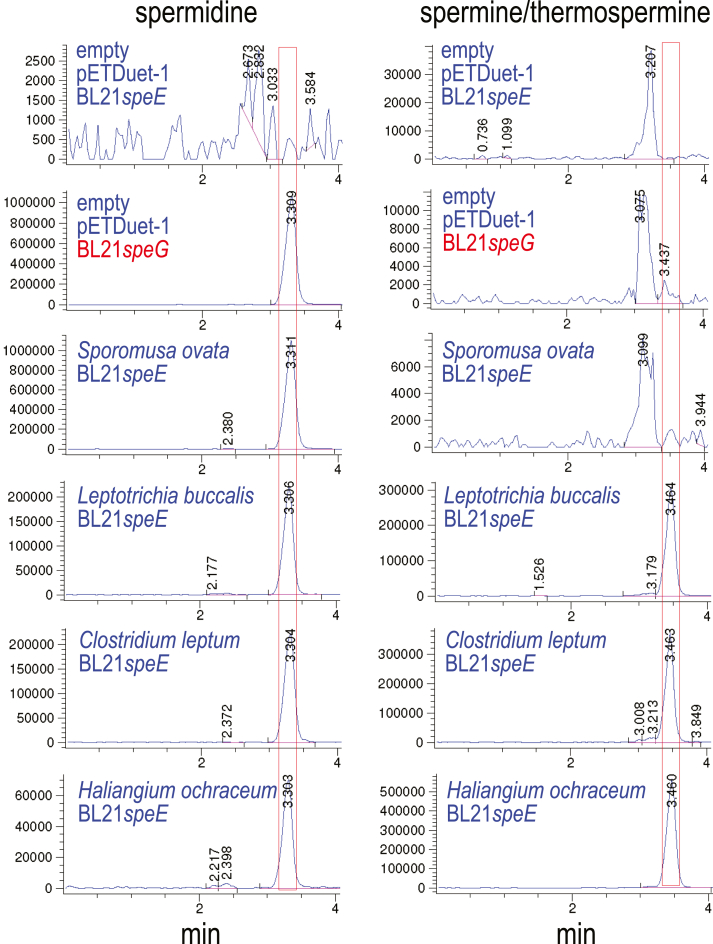
**Expression of diverse aminopropyltransferases in *E. coli* BL21*speE*.** Aminopropyltransferases from genomes also encoding CASDH/CASDC homologs were expressed from pETDuet-1 in the spermidine-void *E. coli* BL21*speE*. Benzoylated polyamines from cell extracts were analyzed by LC-MS. The extracted ion chromatograms for tribenzoylated spermidine (457.94:458.94) and tetrabenzoylated spermine/thermospermine (619.02:620.02) are shown. The spermidine and spermine/thermospermine content of BL21*speG* is also shown for comparison. The y-axis represents arbitrary units of ion intensity, and all samples were grown and analyzed in parallel. CASDC, carboxyspermidine decarboxylase; CASDH, carboxyspermidine dehydrogenase.

We searched for other genomes that encode both CASDH/CASDC and AdoMetDC/APT homologs and selected APT-encoding genes from *Sporomusa ovata* (a diderm Bacillota species), *Leptotrichia buccalis* (Fusobacteriota), *Clostridium leptum* (a monoderm Bacillota species prominent in the gut microbiota), and *Haliangium ochraceum* (Myxococcota). Each APT gene was expressed in the spermidine-void BL21*speE* background, and benzoylated spermidine and Spm/Tspm were detected by LC-MS (Fig. 5). All analyzed APTs produced spermidine, but the *S. ovata* APT was approximately 5-fold more efficient than the *L. buccalis* and *C. leptum* APTs and 15-fold more efficient than the *H. ochraceum* APT. In contrast, the *S. ovata* APT did not produce any detectable Spm/Tspm, whereas all other APTs did, with the *H. ochraceum* APT being the most efficient. We then investigated whether the APTs produced Spm, Tspm, or both, using LC-MS/MS of the benzoylated polyamines (Table 4). This analysis revealed that the *D. desulfuricans*, *L. buccalis*, *C. leptum,* and *H. ochraceum* APTs are highly specific for Spm biosynthesis, with at least four orders of magnitude less Tspm produced. Along with *C. leptum*, other prominent members of the human gut microbiome encode both CASDH/CASDC and AdoMetDC/APT homologs, including *Eubacterium siraeum*, *Bacteroides capillosus,* and *Blautia hansenii* (30). Our data suggests that the APT in these species is likely to be a Spm synthase and indicates a further source of Spm in the gastrointestinal tract.

**Table 4:** LC-MS/MS analysis of spermine and thermospermine production by expression of aminopropyltransferases in *E. coli* BL21*speE and* BL21*speG*

Experimental group/species	AUP 11.37 min (Spm)	AUP 10.69 min (Tspm)
*Group 1*, expression in *E. coli* BL21*speG*		
Empty pETDuet-1	3.38 × 10^4^	2.04 × 10^3^
*Deferribacter desulfuricans* APT	5.96 × 10^8^	5.21 × 10^3^
*Group 2*, expression in *E. coli* BL21*speE*		
Empty pETDuet-1	1.77 × 10^4^	ND
*Sporomusa ovata* APT	1.11 × 10^5^	ND
*Leptotrichia buccalis* APT	6.94 × 10^8^	2.28 × 10^4^
*Clostridium leptum* APT	5.81 × 10^8^	3.40 × 10^4^
*Haliangium ochraceum* APT	7.82 × 10^8^	1.52 × 10^4^

Aminopropyltransferases (APT) expressed from pETDuet-1. Strains in individual groups grown and analyzed together. Shown are AUP values for tetrabenzoylated spermine and thermospermine.

AUP, area under the peak (with elution time); ND, not detected; *speE*, gene deletion of spermidine synthase; *speG*, gene deletion of spermidine *N*-acetyltransferase; Spm, spermine; Tspm, thermospermine.

### Spermidine and Spm biosynthesis via N^1^-aminopropylagmatine

**Figure 6:**
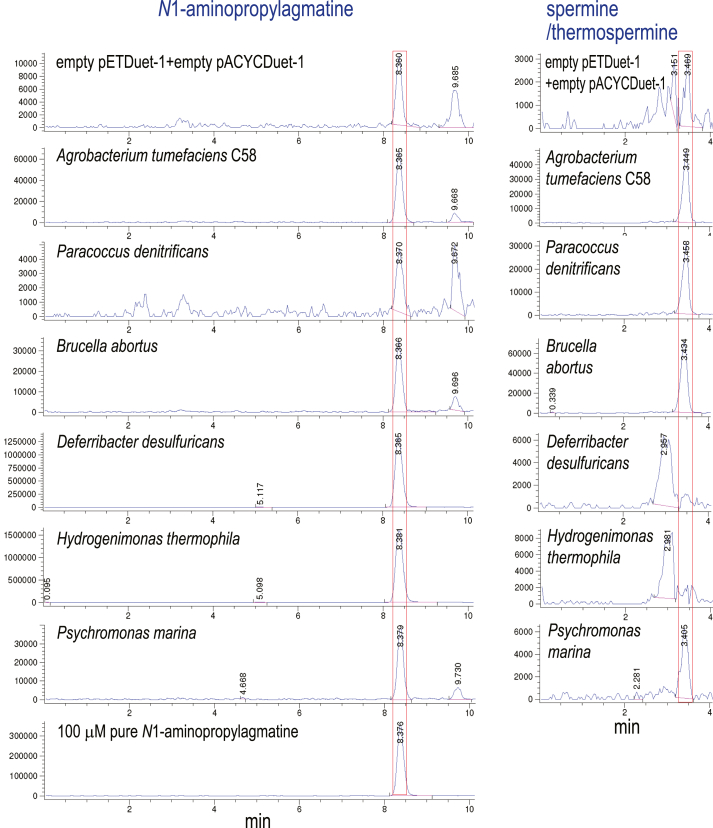
**Expression of diverse CASDH/CASDC pairs in *E. coli* BL21*speB*.** CASDH-encoding open reading frames were expressed from pETDuet-1 and CASDC from pACYCDuet-1 in *E. coli* BL21*speB* grown with 300 μM L-arginine. Benzoylated polyamines from cell extracts were analyzed by LC-MS. Shown are the extracted ion chromatograms for tetrabenzoylated *N*^1^-aminopropylagmatine (605:606) and tetrabenzoylated spermine/thermospermine (619.02:620.02). The y-axis represents arbitrary units of ion intensity, and all samples were grown and analyzed in parallel. CASDC, carboxyspermidine decarboxylase; CASDH, carboxyspermidine dehydrogenase.

We were curious why the *D. desulfuricans* and *H. thermophila* CASDH homologous genes, when expressed alone, did not produce significant carboxyspermidine in BL21*speD*, but coexpression of the CASDH with corresponding CASDC produced a large amount of spermidine. We suspected that these CASDHs might produce carboxyaminopropylagmatine instead of carboxyspermidine, *i.e.*, using agmatine rather than putrescine as a substrate, shown recently for some CASDH homologs (37). The CASDH/CASDC pairs from *A. tumefaciens*, *P. denitrificans*, *B. abortus*, *D. desulfuricans*, *H. thermophila,* and *P. marina* were coexpressed in BL21*speB* grown with 300 μM L-arginine. Benzoylated polyamines were analyzed by LC-MS to detect *N*^1^-aminopropylagmatine and Spm/Tspm (Fig. 6). The *D. desulfuricans* and *H. thermophila* CASDH/CASDC pairs produced approximately 125 to 150 times more *N*^1^-aminopropylagmatine than the native *E. coli* spermidine synthase, whereas the *A. tumefaciens*, *P. denitrificans*, *B. abortus,* and *P. marina* CASDH/CASDC pairs produced at least 20-fold less *N*^1^-aminopropylagmatine than the *D. desulfuricans* and *H. thermophila* enzymes. The low level of *N*^1^-aminopropylagmatine production by the *A. tumefaciens*, *P. denitrificans,* and *B. abortus* enzymes was not due to lack of enzymatic function as these enzymes produced Spm/Tspm in the same samples (Fig. 6). We then compared *N*^1^-aminopropylagmatine production by coexpression of the CASDH/CASDC homologs of *D. desulfuricans* and *H. thermophila* with its production by APTs that we previously showed to be *N*^1^-aminopropylagmatine synthases (13). LC-MS/MS analysis of benzoylated polyamines indicated that the CASDH/CASDC pathway is seemingly more efficient than the AdoMetDC/APT pathway for *N*^1^-aminopropylagmatine production (Table 5).

**Table 5:** LC-MS/MS analysis of *N*^1^-aminopropylagmatine production by coexpression of carboxyspermidine dehydrogenase and decarboxylase or aminopropyltransferases in *E. coli* BL21*speB* grown with 2 mM L-arginine

Species	AUP 21.0 min (*N*^1^-APAgm)
*Coexpression in E. coli BL21speB*	
Empty pETDuet-1 + pACYCDuet-1	6.75 × 10^4^
*Deferribacter desulfuricans* CASDH+CASDC	1.41 × 10^7^
*Hydrogenobacter thermophila* CASDH+CASDC	7.86 × 10^6^
*Psychromonas marina* CASDH + CASDC	6.69 × 10^4^
*Single gene expression in E. coli BL21speB*	
Empty pETDuet-1	4.72 × 10^3^
*Desulfarculus baarsii* Spm synthase	1.15 × 10^4^
*Desulfarculus baarsii N*^1^-APAgm/Spd synthase	1.56 × 10^6^
*Thermococcus kodakarensis N*^1^-APAgm/Spd synthase	6.49 × 10^6^
*Microcystis aeruginosa N*^1^-APAgm/Spd synthase	1.53 × 10^6^
*Thermosyntropha lipolytica N*^1^-APAgm/Spd synthase	1.99 × 10^6^

CASDH expressed from pETDuet-1, CASDC from pACYCDuet-1. All strains were grown and analyzed together. Shown are AUP values for tetrabenzoylated *N*1-aminopropylagmatine daughter ion 218.1 Da (precursor ion 604.597 Da).

AUP, area under the peak (with elution time); *N*^1^-APAgm, *N*^1^-aminopropylagmatine; ND, not detected; *speB*, gene deletion of agmatine ureohydrolase; Spd, spermidine; Spm, spermine.

**Figure 7:**
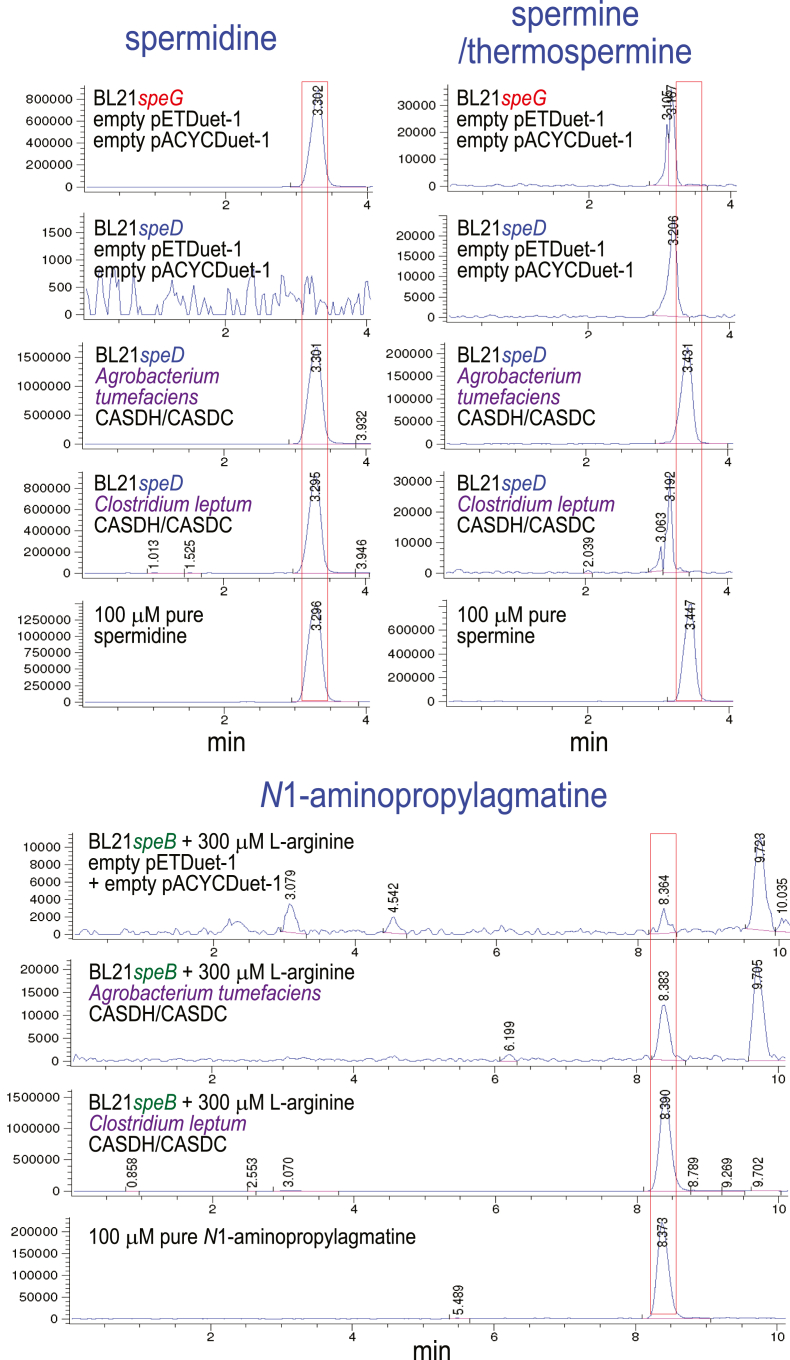
**Expression of CASDH/CASDC pairs from *Agrobacterium tumefaciens* and *Clostridium leptum* in *E. coli* BL21*speD* and BL21*speB* .** Carboxyspermidine dehydrogenase (CASDH)-encoding open reading frames were expressed from pETDuet-1 and carboxyspermidine decarboxylase (CASDC) from pACYCDuet-1 in spermidine-void BL21*speD* and in *E. coli* BL21*speB* grown with 300 μM L-arginine. Benzoylated polyamines from cell extracts were analyzed by LC-MS. Shown are the extracted ion chromatograms for tribenzoylated spermidine (457.94:458.94), tetrabenzoylated spermine/thermospermine (619.02:620.02), and tetrabenzoylated *N*^1^-aminopropylagmatine (605:606). The spermidine and spermine/thermospermine content of BL21*speG* is also shown for comparison. The y-axis represents arbitrary units of ion intensity, and all samples were grown and analyzed in parallel.

To assess whether generally, in genomes encoding CASDH/CASDC and AdoMetDC/APT, the CASDH/CASDC pathway was likely to synthesize spermidine *via N*^1^-aminopropylagmatine, we coexpressed the CASDH/CASDC-encoding homologs from *C. leptum* in BL21*speD* and in BL21*speB*. As a comparative example of a *bona fide* carboxyspermidine pathway, we also coexpressed the CASDH/CASDC pair from *A. tumefaciens* in the same *E. coli* strains (Fig. 7). The *A. tumefaciens* CASDH/CASDC pair produced approximately twice as much spermidine in BL21*speD* compared to the *C. leptum* genes, and whereas the *A. tumefaciens* genes produced Spm/Tspm, none was produced by expression of the *C. leptum* CASDH/CASDC. In contrast, the *C. leptum* CASDH/CASDC pair produced approximately 150-fold more *N*^1^-aminopropylagmatine compared to the *A. tumefaciens* CASDH/CASDC (Fig. 7).

The CASDH homologous enzymes encoded by *D. desulfuricans* and *H. thermophila* produce only low levels of carboxyspermidine, whereas, like *C. leptum*, their CASDH/CASDC homologs synthesize a large amount of *N*^1^-aminopropylagmatine. It is therefore likely that these CASDH/CASDC enzymes are actually carboxyaminopropylagmatine dehydrogenases/decarboxylases (CAPADH/CAPADC), as has been shown for the homologous enzymes from the cyanobacterium *Synechocysti*s sp. 6803 (37).

### Correlation of L-ornithine decarboxylase with carboxyspermidine and L-arginine decarboxylase with N^1^-aminopropylagmatine routes

An alanine racemase-fold ODC (38), that produces putrescine directly from ornithine and which is homologous to the human ODC, is encoded by *A. tumefaciens*, *P. denitrificans,* and *B. abortus* [*A*.*t*., GenBank protein acc. no. WP_013761479 (377 a.a.); *P*.*d*., WP_104491843 (385 a.a.); *B*.*a*., WP_002966479 (377 a.a.)]. However, agmatine-producing arginine decarboxylases (ADCs) from the alanine racemase fold (homologous to the *E. coli* ADC/SpeA) are encoded by *D. desulfuricans* and *H. thermophila* [*D*.*d*., WP_013008002 (613 a.a.); *H*.*t*., WP_317066233 (624 a.a.)]. An aspartate aminotransferase-fold ADC, homologous to the *Bacillus subtilis* ADC/SpeA, is encoded by *C. leptum* (EDO61987). The lack of encoded ADC, and therefore of agmatine, in *A. tumefaciens*, *P. denitrificans,* and *B. abortus*, precludes the formation of *N*^1^-aminopropylagmatine and favors formation of carboxyspermidine from putrescine by CASDH/CASDC in these species.

**Figure 8:**
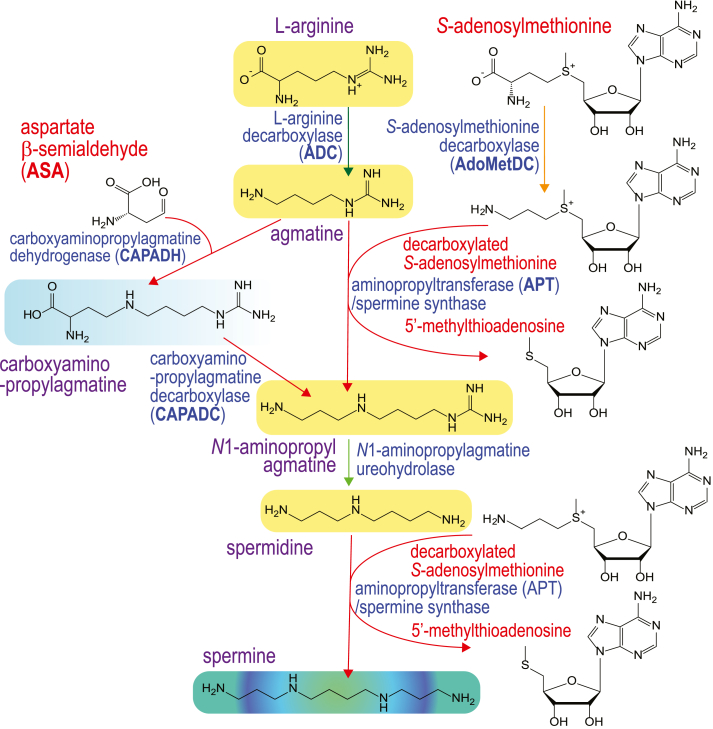
**A hybrid pathway for spermine biosynthesis from agmatine *via* carboxyaminopropylagmatine, *N*^1^-aminopropylagmatine, and spermidine.** Aspartate β-semialdehyde-dependent formation of *N*^1^-aminopropylagmatine *via* carboxyaminopropylagmatine converges on decarboxylated S-adenosylmethionine-dependent formation of *N*^1^-aminopropylagmatine for production of spermidine and spermine. CASDC, carboxyspermidine decarboxylase; CASDH, carboxyspermidine dehydrogenase.

Based on our data for the activity of the *D. desulfuricans* and *C. leptum* CAPADH/CAPADC and AdoMetDC/APT, we propose a hybrid pathway for Spm biosynthesis initiating from L-arginine and proceeding *via* agmatine, L-carboxyaminopropylagmatine, *N*^1^-aminopropylagmatine, and spermidine, utilizing dcAdoMet and ASA for provision of aminopropyl groups (Fig. 8). In this proposed pathway, *N*^1^-aminopropylagmatine can be produced by both CAPADH/CAPADC and by AdoMetDC/APT, but the bulk of spermidine production will be by CAPADH/CAPADC. The second aminopropylation step from spermidine to Spm is performed only by AdoMetDC/APT

### CASDH not CASDC determines substrate specificity

**Figure 9:**
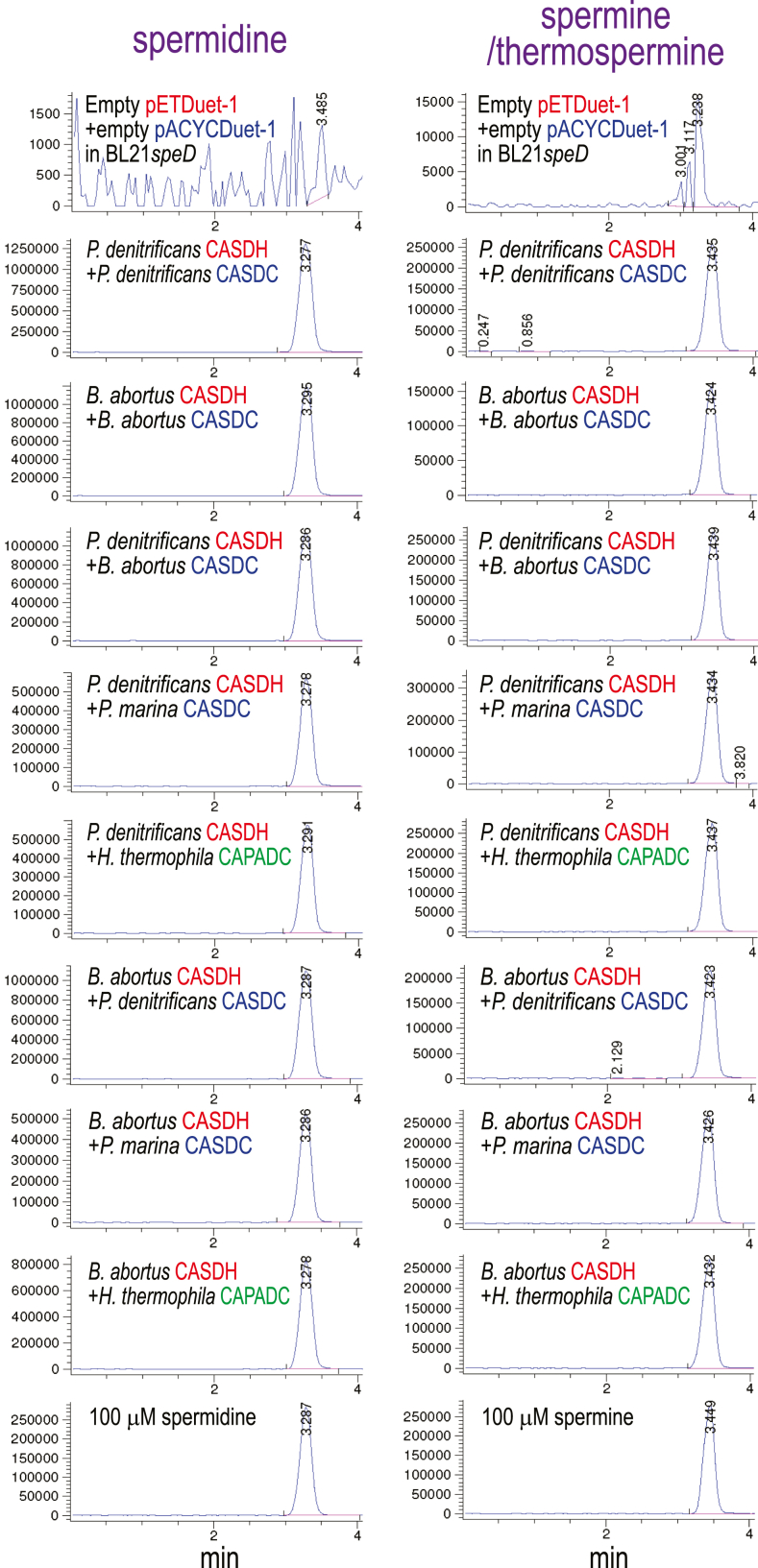
**Expression of diverse CASDH/CASDC and CASDH/CAPADC pairs in *E. coli* BL21*speD*.** Carboxyspermidine dehydrogenase (CASDH)-encoding open reading frames were expressed from pETDuet-1 and carboxyspermidine decarboxylase (CASDC) or carboxyaminopropylagmatine decarboxylase (CAPADC) from pACYCDuet-1 in spermidine-void BL21*speD*. Benzoylated polyamines from cell extracts were analyzed by LC-MS. The extracted ion chromatograms for tribenzoylated spermidine (457.94:458.94) and tetrabenzoylated spermine/thermospermine (619.02:620.02) are shown. The y-axis represents arbitrary units of ion intensity, and all samples were grown and analyzed in parallel.

CASDH/CASDC and CAPADH/CAPADC pairs exhibit very different substrate specificity toward putrescine and agmatine. The *A. tumefaciens* and *P. denitrificans* CASDH/CASDC are approximately 100-fold more efficient at producing carboxyspermidine in BL21*speD* than the *D. desulfuricans* and *H. thermophila* CAPADH/CAPADC (Table 2). In contrast, *D. desulfuricans* and *H. thermophila* CAPADH/CAPADC are approximately more than 100-fold more efficient than the *A. tumefaciens* and *P. denitrificans* CASDH/DC at producing *N*^1^-aminopropylagmatine in BL21*speB* (Fig. 6). We wondered whether substrate specificity is provided by both CASDH/CAPADH and CASDC/CAPADC or only by CASDH/CAPADH. Matching or mixed pairs of CASDH/CASDC and CASDH/CAPADC genes were expressed in BL21*speD* and the accumulation of spermidine and Spm/Tspm assessed by LC-MS of the benzoylated polyamines. When the carboxyspermidine-producing CASDHs from *P. denitrificans* and *B. abortus* were coexpressed with the CAPADC from *H. thermophila*, which decarboxylates carboxyaminopropylagmatine, the level of spermidine produced was reduced by only approximately 20 to 50% compared to the matching CASDC, suggesting a minor contribution of CASDC to substrate specificity (Fig. 9). For Spm/Tspm production by *P. denitrificans* and *B. abortus* CASDH, coexpression with the *H. thermophila* CAPADC maintained or increased their levels relative to matched coexpression of the corresponding CASDCs. Similarly, coexpression of the *P. denitrificans* and *B. abortus* CASDHs with the *P. marina* CASDC (which produces only a trace of Spm/Tpsm) does not decrease Spm/Tspm levels. This again suggests that the decarboxylases CASDC/CAPADC do not have a significant role in substrate specificity, which appears to be controlled primarily by CASDH.

## Discussion

We have identified for the first time an ASA-dependent pathway for Spm/Tspm biosynthesis. In the α-proteobacteria *A. tumefaciens*, *B. abortus* and *P. denitrificans*, the ODC route, which produces putrescine directly from L-ornithine, is the only pathway present for polyamine biosynthesis. Heterologous production of carboxyspermidine in *E. coli* by the *A. tumefaciens* and *P. denitrificans* CASDHs confirms that putrescine rather than agmatine is the initial precursor for spermidine biosynthesis. The subsequent production of C-Spm/C-Tspm from spermidine by the same CASDHs corroborates the existence of a novel ASA-dependent pathway for Spm/Tspm biosynthesis, initiating from putrescine. Unlike bacterial dcAdoMet-dependent APTs that produce Spm or Tspm from putrescine or agmatine (13), the CASDH/CASDC pathway produces less Spm/Tspm than spermidine. In native *P. denitrificans* and *A. tumfaciens*, Spm/Tspm is not accumulated during normal growth but is induced by the production of spermidine-containing parabactin siderophore in *P. denitrificans* (27) or by inhibition of ODC in *A. tumfaciens* (26). This suggests that a reduction in the level of spermidine induces Spm/Tspm accumulation and that spermidine may inhibit Spm/Tspm formation by CASDH. Reduction of spermidine levels may then allow carboxyaminopropylation of spermidine by CASDH to produce C-Spm/C-Tspm, followed by decarboxylation by CASDC to produce Spm/Tspm. The purified recombinant *A. tumefaciens* CASDH protein was previously shown to form carboxyspermidine from ASA and putrescine, but spermidine was not tested as a substrate (39).

In the α-proteobacterium *Rhodothalassium salexigens*, aminopropylated homospermidine (*N*^1^-aminopropylhomospermidine) is the major polyamine (40). A homospermidine synthase is encoded by *A. tumefaciens* and *B. abortus*, and we have shown that the corresponding CASDH/CASDC genes from these species can heterologously aminopropylate homospermidine to form *N*^1^-aminopropylhomospermidine. The *P. denitrificans* CASDH/CASDC proteins can also aminopropylate homospermidine, but *P. denitrificans* does not encode homospermidine synthase, suggesting this activity is an inherent property of the Spm/Tspm-forming CASDH/CASDC pathway. In principle, *A. tumefaciens* and *B. abortus* should be able to synthesize three different tetraamines: *N*^1^-aminopropylhomospermidine, Spm, and Tspm.

The *D. desulfuricans* CASDH/CASDC proteins synthesized spermidine but not Spm/Tspm; however, the genome also encoded AdoMetDC and an APT. We showed that the APT was a specific Spm synthase and that it could synthesize Spm in the absence of native spermidine. Furthermore, the *D. desulfuricans* APT very efficiently aminopropylates agmatine, indicating that this APT likely produces Spm from agmatine *via N*^1^-aminopropylagmatine and spermidine. The *D. desulfuricans* CASDH/CASDC proteins are also very efficient at aminopropylating agmatine and produce spermidine from agmatine *via N*^1^-aminopropylagmatine. Therefore, for *D. desulfuricans*, agmatine and *N*^1^-aminopropylagmatine are common biosynthetic intermediates between the ASA- and dcAdoMet-dependent polyamine biosynthetic pathways. This is the first biochemical demonstration of a potentially hybrid ASA-/dcAdoMet-dependent pathway, although we have previously noted that some firmicute (Bacillota) and Bacteroidetes (Bacteroidota) species encode both pathways (30). We then showed that in phylogenetically diverse bacteria that encode both CASDH/CASDC and AdoMetDC/APT, the APT encodes a specific Spm synthase (*L. buccalis*, *C. leptum,* and *H. ochraceum*). We also confirmed that the twin pathways from *C. leptum* behave in exactly the same way as the *D. desulfuricans* pathways, with the CASDH/CASDC pathway synthesizing spermidine from agmatine and *N*^1^-aminopropylagmatine, and the APT synthesizing Spm from agmatine, *N*^1^-aminopropylagmatine, and spermidine. It remains to be shown whether the ASA- and dcAdoMet-dependent pathways interact through shared intermediates. Why would bacteria encode two biosynthetically different pathways to synthesize spermidine and Spm? Parallel pathways might allow Spm biosynthesis to be independent of spermidine biosynthesis; however, shared biosynthetic intermediates would also allow a more efficient production of Spm.

There appear to be distinct carboxyaminopropylation pathways: those acting on putrescine and spermidine (*P. denitrificans*, *A. tumefaciens,* and *B. abortus*) and those acting agmatine (*D. desulfuricans*, *H. thermophila*, and *C. leptum*). It is likely that the *P. marina* CASDH/CASDC homologs act on 1,3-diaminopropane in the native bacterium to produce norspermidine, since this species encodes L-2,4-diaminobutyrate: 2-ketoglutarate 4-aminotransferase (WP_284203354; 469 a.a.) and L-2,4-diaminobutyrate decarboxylase (WP_284203353; 491 a.a.) to produce 1,3-diaminopropane from ASA (41). Substrate specificity of the CASDH/CASDC homologs appears to be primarily dependent on the CASDH step. The CAPADC of *H. thermophila* and the putative carboxynorspermidine decarboxylase of *P. marina* appear to be as efficient as the *P. denitrificans* and *B. abortus* carboxyspermidine/C-Spm/C-Tspm decarboxylases in producing Spm/Tspm. However, they are approximately 2-fold less efficient at producing spermidine. Although CASDH/CANSDH/CAPADH protein sequences may be able to help distinguish carboxyspermidine from carboxynorspermidine and carboxyaminopropylagmatine pathways, it is probably more effective to determine whether ADC (agmatine), ODC (putrescine), or L-2,4-diaminobutyrate: 2-ketoglutarate 4-aminotransferase/L-2,4-diaminobutyrate decarboxylase (1,3-diaminopropane) are encoded by a given genome. The ASA-dependent pathway consisting of CASDH/CASDC homologs has diversified during evolution to produce norspermidine (29), *N*^1^-aminopropylagmatine (37), and as we have shown here, spermidine and Spm/Tspm.

## Experimental procedures

### Chemicals and reagents

Homospermidine was a kind gift from Dr Patrick Woster. Tspm (cat. no. Sc-472594B) was obtained from Santa Cruz Biotechnology. Agmatine (A7127-5G), putrescine (P5780-5G), norspermidine (I1006–100G), spermidine (85580), norspermine (404810-5G), and spermine (85605-1G) were obtained from Sigma Aldrich. *N*^1^-aminopropylagmatine was custom synthesized by WuXi AppTec. Expression plasmids pETDuet-1 and pACYCDuet-1 were purchased from Novagen. Genes with *E. coli*-optimized codons were synthesized by GenScript. All proteins analyzed in this study are described in Table S1.

### Bacterial strains, growth, and gene expression

Construction of BL21-derived strains was described previously: BL21*speB*, agmatine ureohydrolase gene deletion (13); BL21*speD*, AdoMetDC deletion (42); BL21*speE*, spermidine synthase gene deletion (43); and BL21*speG*, spermidine *N*-acetyltransferase deletion (34). Strains of *E. coli* were grown twice in 2 ml of liquid, polyamine-free M9 minimal medium, at 37 °C overnight. A 1.0 ml aliquot of culture was then centrifuged, the supernatant discarded, and cells resuspended in 10 ml M9 medium and grown at 37 °C to *A*_600_ = 0.5. Gene expression from pETDuet-1 and pACYCDuet-1 was induced by addition of 0.5 mM isopropyl-β-d-thiogalactopyranoside, and cultures were maintained at 16 °C, overnight. Cells were then centrifuged, and polyamines extracted.

### Polyamine extraction and benzoylation reaction

Cultures of *E. coli* BL21 strains were pelleted by centrifugation and washed three times by resuspension in PBS. Repelleted cells were resuspended in 200 μl of lysis buffer (100 mM MOPS pH 8.0, 50 mM NaCl, 20 mM MgCl_2_), frozen in liquid nitrogen, and thawed at 37 °C, and this was repeated three times. To the lysate was added 60 μl of 40% trichloroacetic acid and after thorough mixing, kept on ice for 10 min. Cellular debris was pelleted by centrifugation at 4 °C, and the supernatant transferred to a new tube for benzoylation, which improves polyamine chromatographic separation and detection. The mass of the benzoyl moiety is 105 Da, and spermidine is benzoylated on three amine positions, *N*^1^-aminopropylagmatine, Spm, and Tspm and norspermine on four. To 200 μl of the cell supernatant containing extracted polyamines, 1 ml of 2 M NaOH was added followed by 10 μl of benzoyl chloride, and this mixture was vigorously vortexed for 2 min and left at room temperature for 1 h. Two milliliter of saturated NaCl was added to this mixture, followed by further mixing for 2 min, and then 2 ml of diethyl ether added, vortexing for another 2 min and left at room temperature for 30 min. The upper layer of diethyl ether containing the polyamines was transferred to a new tube and kept in a chemical hood until fully evaporated.

### Liquid chromatography-mass spectrometry

Samples of benzoylated cell extract were run on an Agilent 1290 Infinity HPLC system fitted with an Eclipse XDB-C18 column (4.6 × 150 mm, 5 μm particle size), coupled to an Agilent 6130 quadrapole ESI mass spectrometer run in positive mode, employing a scan range of 100 to 1100 m/z. A flow rate of 0.5 ml/min at 20 °C was used for the liquid chromatography stage, with a 5 μl injection volume, employing a gradient elution with aqueous acetonitrile containing 0.1% formic acid.

### LC-MS/MS separation and quantification of tetrabenzoylated spermine, Tspm, and N^1^-aminopropylagmatine

High-performance liquid chromatography conditions: reverse phase chromatography was performed using an ACE 3 C18-PFP 150 × 4.6 mm, 3 μm HPLC column (Mac-Mod). Column temperature, sample injection volume, and flow rate was set to 30 °C, 5 μl, and 0.8 ml/min respectively. HPLC conditions were as follows: Solvent A: water with 0.1% formic acid (v/v), Optima LC/MS Grade. Solvent B: acetonitrile with 0.1% formic Acid (v/v), Optima LC/MS Grade: 40% B, 0 to 13 min; 5% B, 15 to 18 min; 95% B, 20 to 23 min; 40% B, 24 to 30 min. Total run time 30 min. Data were processed by SCIEX MultiQuant 3.0.3 software (AB Sciex) with relative quantification based on the peak area of each metabolite. Targeted mass spectrometric analyses were performed on an AB Sciex QTRAP 6500+ mass spectrometer equipped with an ESI ion spray source. The ESI source was used in positive ion mode. Ion source conditions in the positive mode were as follows: Ion Source Gas 1, 70 p.s.i.; Ion Source Gas 2, 65 p.s.i.; Curtain gas, 45 p.s.i.; ion spray voltage, 5500 V; and source temperature, 550 °C. Data acquisition was performed in multiple reaction monitoring (MRM) mode. Three diagnostic MRM transitions in the positive mode for tetrabenzoylated spermine (elution at 11.50 min) and Tspm (10.63 min) were obtained, and three MS ion transitions Q1/Q3, 619.228/497.2; 619.228/162 and 619.228/77 were monitored. The MS transition of Q1/Q3, 619.228/497.2, was used as the quantifier ion while 619.228/162 and 619.228/77 were used as the qualifier ions. Three diagnostic MRM transitions in the positive mode for tetrabenzoylated N1-aminopropylagmatine (elution at 21.0 min) were obtained, and three MS ion transitions Q1/Q3, 604.597/233; 604.597/218.1 and 604.597/77 were monitored. The mass spectrometer was coupled to a Shimadzu HPLC (Nexera X2 LC-30AD) and was controlled by Analyst 1.7 software.

### High-resolution LCMS analysis of carboxyspermidine and C-Spm/C-Tspm

Untargeted mass spectrometric analyses were performed on a Sciex TripleTOF 6600 system (AB SCIEX) equipped with an electrospray ionization source used in the positive ionization mode and configured as follows: ion source gas 1, 65 p.s.i; ion source gas 2, 60 p.s.i; curtain gas, 25 p.s.i.; source temperature, 550 °C; and ion spray voltage floating, +5500 V. TOF-MS mode (full scan) and information-dependent acquisition mode (product ion scan) were utilized to collect MS and MS/MS data, respectively. For TOF-MS scans, the mass range was from m/z 60 to 1000, and for product ion scans, the mass range was from m/z 60 to 1000. The collision energy was set at 30 V (+), and collision energy spread was ±15 V. Accumulation time was 0.25 s for TOF-MS scans and 0.06 s for product ion scans. The instrument was automatically calibrated using a calibration delivery system injected in APCI positive calibration solution every five samples. The mass spectrometer was coupled to a Shimadzu HPLC (Nexera X2 LC-30AD), and system was controlled by Analyst TF 1.8.1 software (Sciex). LC-MS separation conditions: reverse phase chromatography was performed using an ACE 3 C18-PFP 150 × 4.6 mm, 3 μm HPLC column (Mac-Mod). Column temperature, sample injection volume, and flow rate was set to 30 °C, 5 μl, and 0.5 ml/min, respectively. HPLC conditions were as follows: Solvent A: water with 0.1% formic acid (v/v), Optima LC/MS Grade. Solvent B: acetonitrile with 0.1% formic acid (v/v), Optima LC/MS Grade: 2% B, 0 to 2 min; 90% B, 5 to 16 min; 2% B, 17 to 30 min. Total run time 30 min. This system does not distinguish between the same mass isomers C-Spm and C-Tspm.

### Gene identification

Homologs of CASDH and CASDC were found using BLASTP analysis of specific genomes with biochemically confirmed probe protein sequences. Identification of genomes encoding CASDH/CASDC homologs and AdoMetDC/APT homologs was achieved by using TBLASTN of all eubacterial genomes, using a concatenated CASDH/CASDC/AdoMetDC/APT protein sequence.

## Data availability

All data presented are contained within the article.

## Supporting information

This article contains supporting information.

## Conflict of interest

The authors declare no conflict of interests with the contents of this article.
