# Supplementary material for: New routes for spermine biosynthesis
Source: J Biol Chem. 2025 Mar 10;301(4):108390. doi: 10.1016/j.jbc.2025.108390 (PMC11999265; doi:10.1016/j.jbc.2025.108390)
Supplement: Supporting Information [file mmc1.pdf]

# Supporting Information

# New routes for spermine biosynthesis

Bin Li[1], Hamid R. Baniasadi[1], Jue Liang[1], Margaret A. Phillips[1] and Anthony J. Michael[1*]

[1]Department of Biochemistry, UT Southwestern Medical Center, Dallas, Texas, USA

[*]For correspondence: Anthony J. Michael, anthony.michael@utsouthwestern.edu

**Table S1. Proteins analyzed in this study**

| Species (Phylum) | **Protein** [GenBank acc. no.] | Size (a.a.) |
|---|---|---|
| ***Paracoccus denitrificans* (α-Proteobacteria)** | **CASDH** [WP_011749229] | 407 |
| ***Paracoccus denitrificans* (α-Proteobacteria)** | **CASDC** [WP_011749230] | 368 |
| ***Agrobacterium tumefaciens* (α-Proteobacteria)** | **CASDH** [WP_010973612] | 412 |
| ***Agrobacterium tumefaciens* (α-Proteobacteria)** | **CASDC** [WP_010973611] | 365 |
| ***Brucella abortus* (α-Proteobacteria)** | **CASDH** [WP_002963500] | 413 |
| ***Brucella abortus* (α-Proteobacteria)** | **CASDC** [WP_002963499] | 365 |
| ***Deferribacter desulfuricans* (Deferribacterota)** | **CAPADH** [WP_013008001] | 406 |
| ***Deferribacter desulfuricans* (Deferribacterota)** | **CAPADC** [WP_013008000] | 394 |
| ***Deferribacter desulfuricans* (Deferribacterota)** | **APT** [WP_013008000] | 277 |
| ***Clostridium leptum* (Bacillota)** | **CAPADH** [EDO61991] | 399 |
| ***Clostridium leptum* (Bacillota)** | **CAPADC** [EDO61992] | 376 |
| ***Clostridium leptum* (Bacillota)** | **APT** [EDO61989] | 284 |
| ***Hydrogenimonas thermophila* (ε-Proteobacteria)** | **CAPADH** [WP_317066177] | 399 |
| ***Hydrogenimonas thermophila* (ε-Proteobacteria)** | **CAPADC** [WP_317066176] | 378 |
| ***Psychromonas marina* (γ-Proteobacteria)** | **CANSDH** [WP_284203352] | 405 |
| ***Psychromonas marina* (γ-Proteobacteria)** | **CANSDC** [WP_284203351] | 373 |
| ***Sporomusa ovata* (Bacillota)** | **APT** [WP_021166903] | 278 |
| ***Leptotrichia buccalis* (Fusobacteriota)** | **APT** [WP_015770029] | 280 |
| ***Haliangium ochraceum* (Myxococcota)** | **APT** [WP_012829793] | 283 |

CASDH, carboxyspermidine dehydrogenase; CASDC, carboxyspermidine decarboxylase; CAPADH, carboxyaminopropylagmatine dehydrogenase; CAPADC, carboxyaminopropylagmatine decarboxylase; CANSDH, carboxynorspermidine dehydrogenase; CANSDC, carboxynorspermidine decarboxylase: APT, aminopropyltransferase.

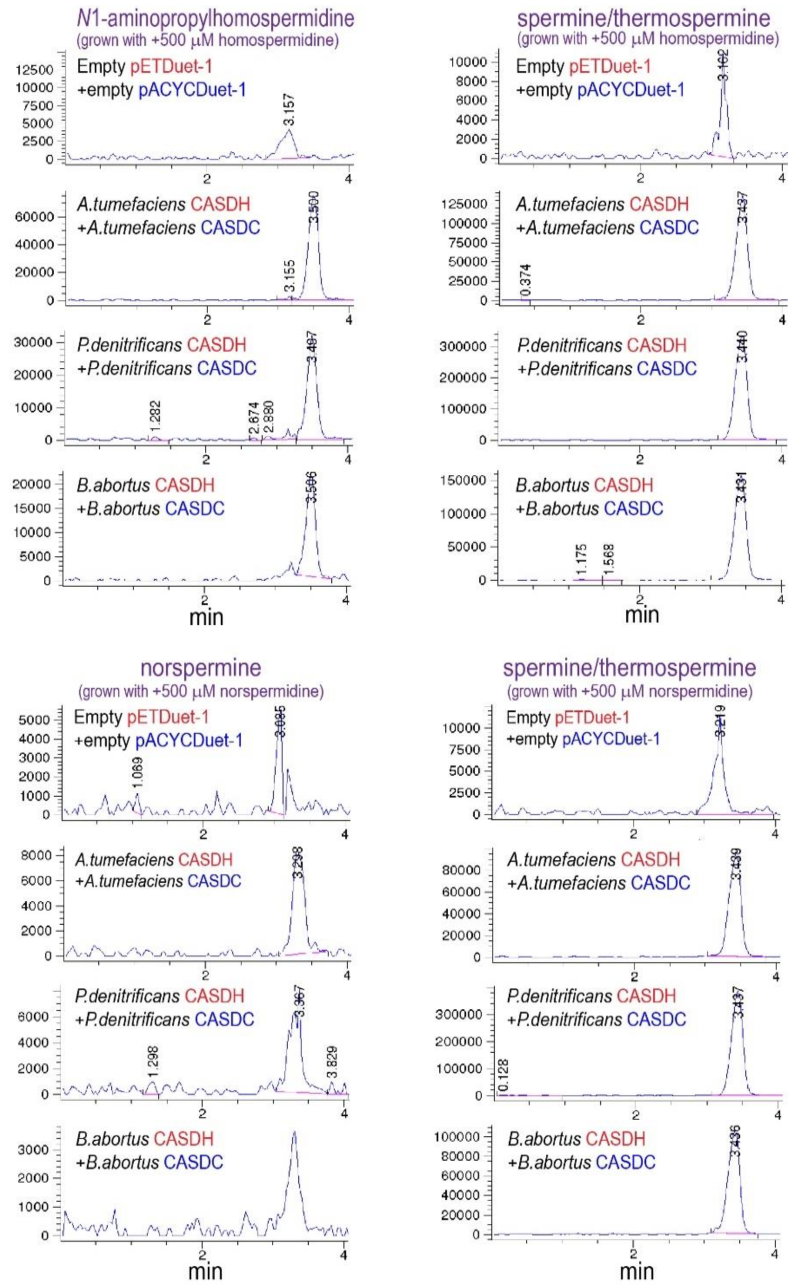


**Figure S1.** ***N*[1]-aminopropylhomospermidine and norspermine production by CASDH/CASDC in *E. coli* BL21*speD*.** Spermidine-void *E.coli* BL21*speD* strains coexpressing CASDH/CASDC were grown with either 500 μM homospermidine or 500 μM norspermidine. Polyamines from cell extracts were benzoylated and analyzed by LC-MS. Shown are the Extracted Ion Chromatograms (EICs) for tetrabenzoylated $N^1$-aminopropylhomospermidine (mass tolerance window 633.04:634.04), tetrabenzoylated norspermine (605:606) and tetrabenzoylated spermine/thermospermine (619.02:620.02). CASDH and CASDC from the indicated species were coexpressed from pETDuet-1 and pACYCDuet-1, respectively, in spermidine-void *E.coli* BL21*speD*. The y-axis represents arbitrary units of ion intensity, and all samples were grown, extracted and analyzed together.

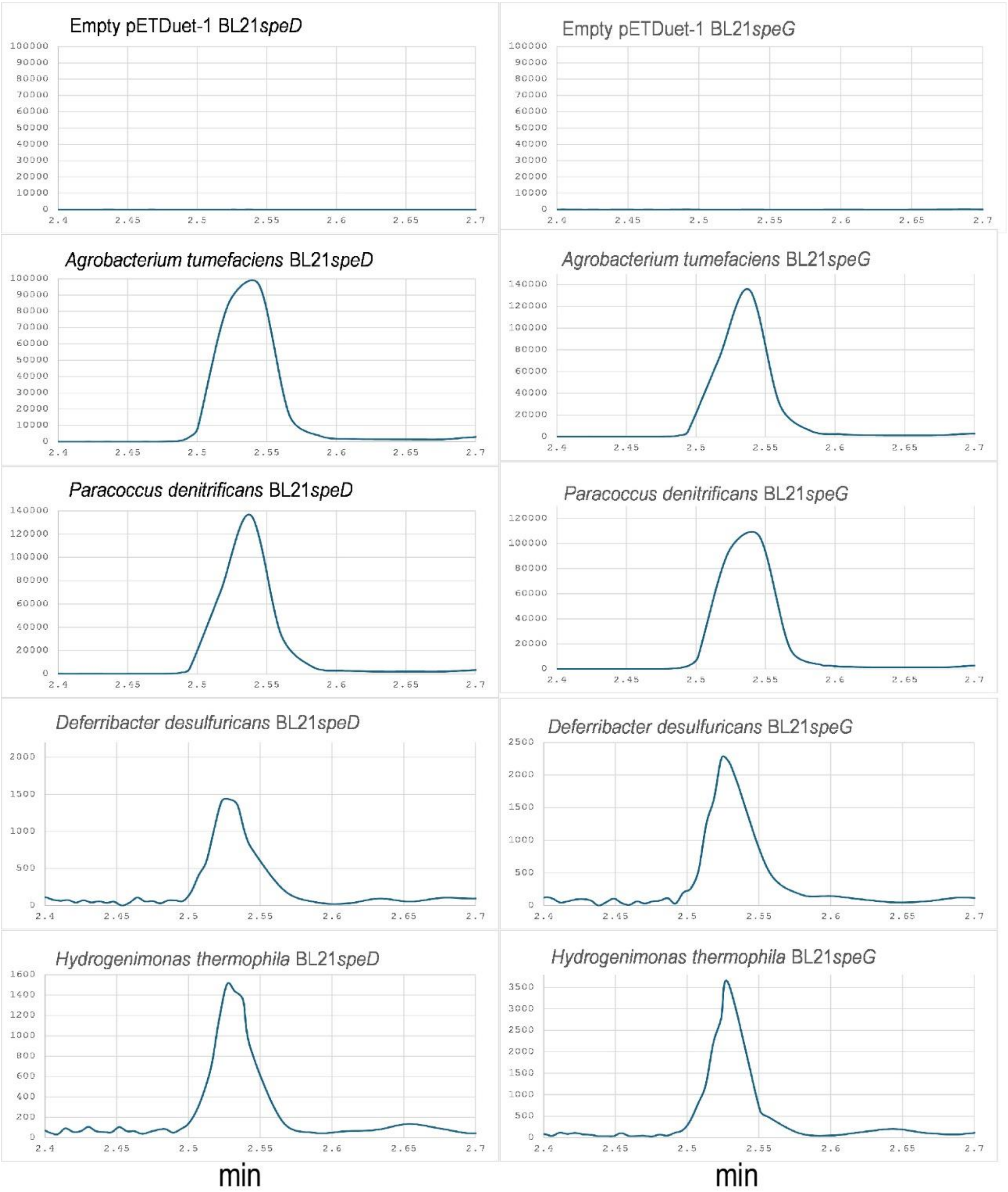


**Figure S2. High Resolution-LCMS detection of underivatized carboxyspermidine after expression of CASDH homologs in *E. coli* BL21*speD* and BL21*speG*.** Shown are the HR-LCMS chromatograms relating to Table 2 in the main text.

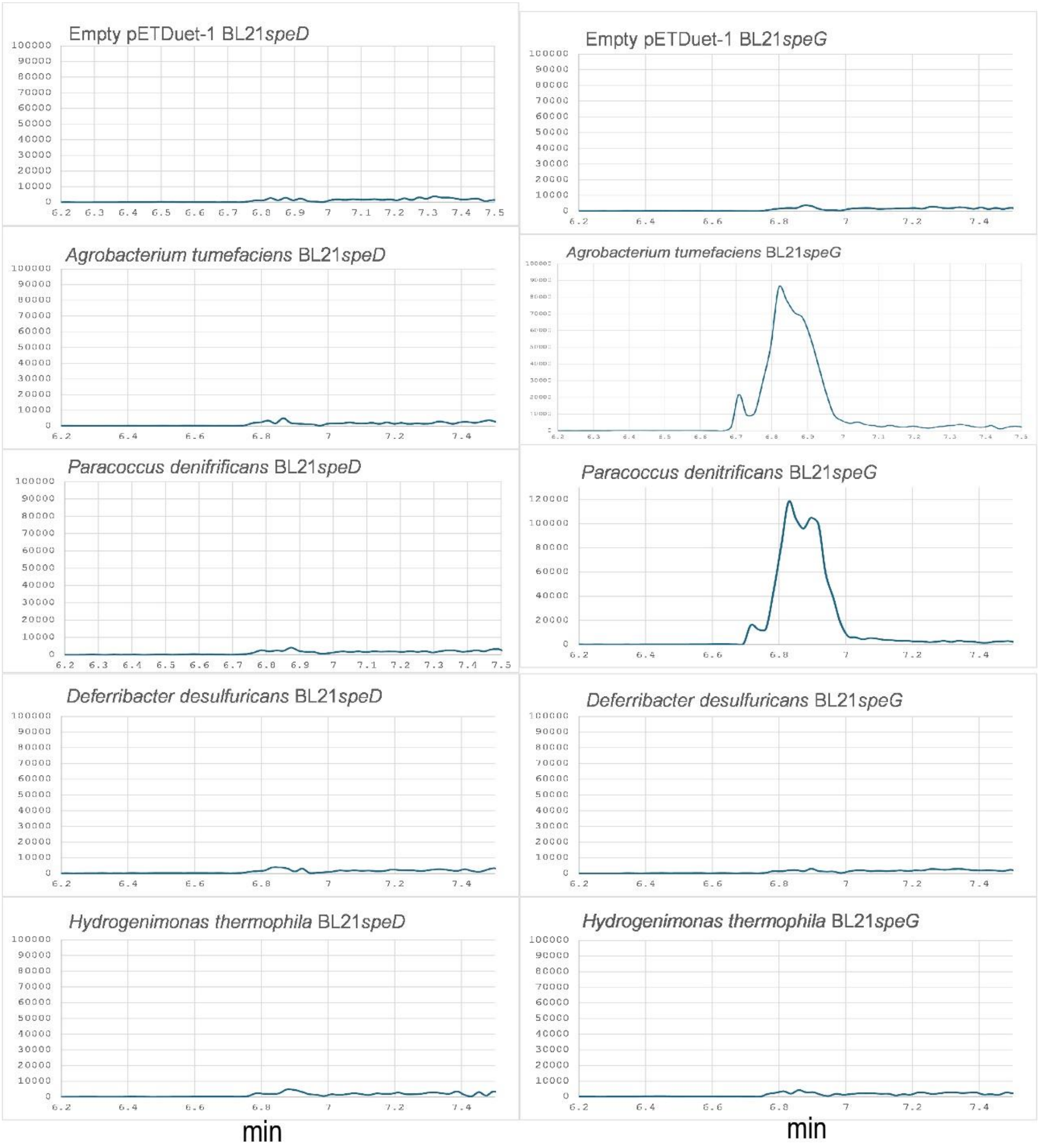


**Figure S3. High Resolution-LCMS detection of underivatized carboxyspermine/ carboxythermospermine after expression of CASDH homologs in *E. coli* BL21*speD* and BL21*speG*.** Shown are the HR-LCMS chromatograms relating to Table 3 in the main text. Note that carboxyspermine and carboxythermospermine are not clearly resolved.
